# Applications of Raman micro-spectroscopy to stem cell technology: label-free molecular discrimination and monitoring cell differentiation

**DOI:** 10.1140/epjti/s40485-015-0016-8

**Published:** 2015-03-24

**Authors:** Adrian Ghita, Flavius C Pascut, Virginie Sottile, Chris Denning, Ioan Notingher

**Affiliations:** School of Physics and Astronomy, University of Nottingham, Nottingham, NG7 2RD UK; School of Medicine, University of Nottingham, Nottingham, NG7 2RD UK

## Abstract

Stem cell therapy is widely acknowledged as a key medical technology of the 21st century which may provide treatments for many currently incurable diseases. These cells have an enormous potential for cell replacement therapies to cure diseases such as Parkinson’s disease, diabetes and cardiovascular disorders, as well as in tissue engineering as a reliable cell source for providing grafts to replace and repair diseased tissues. Nevertheless, the progress in this field has been difficult in part because of lack of techniques that can measure non-invasively the molecular properties of cells. Such repeated measurements can be used to evaluate the culture conditions during differentiation, cell quality and phenotype heterogeneity of stem cell progeny. Raman spectroscopy is an optical technique based on inelastic scattering of laser photons by molecular vibrations of cellular molecules and can be used to provide chemical fingerprints of cells or organelles without fixation, lysis or use of labels and other contrast enhancing chemicals. Because differentiated cells are specialized to perform specific functions, these cells produce specific biochemicals that can be detected by Raman micro-spectroscopy. This mini-review paper describes applications of Raman micro-scpectroscopy to measure moleculare properties of stem cells during differentiation in-vitro. The paper focuses on time- and spatially-resolved Raman spectral measurements that allow repeated investigation of live stem cells in-vitro.

## Introduction

### Tissue engineering and stem cells technology

The capacity of the human body to recover from injuries or diseases is limited and in some situations almost non-existent. The standard medical procedures, which involve surgery and drug based-therapies, have shown limitations in treating many complex conditions. New alternatives to existing medical treatments are required as the incidence of the medical conditions with clinical impact on society in terms of mortality, morbidity and quality of life is continuously increasing. Tissue engineering is an emerging field in modern medicine that aims to grow tissue in laboratories that can be used for replacement of diseased tissue and organs, repair and regeneration. Tissue replacement involves the growth of tissue and organs in-vitro that can be implanted in the human body [1]. The repair involves medical treatment at the biological and molecular level. The main advantages of these approaches are related to an increased availability of grafts and reducing the risks associated with viral infections (e.g. tissue rejection) or negative immunological response [2].

A number of different sources of cells are considered for tissue engineering and regenerative medicine. Mature fully differentiated somatic cells, adult and embryonic stem cells have been used in different applications. The somatic cells extracted from the patients have no risks associated with the integration and immune response, but these cells can be obtained in limited numbers and have low proliferation rates. Adult stem cells are immature undifferentiated cells extracted from the human body, usually bone marrow, and are capable of generating daughter cells. The self-renewal process occurs over the entire lifetime of the biological host. However, the limited number of available adult stem cells in the human body gives rise to impediments in the self-healing capabilities of the human body. Embryonic stem cells (ESCs) are derived from the inner cell mass of mammalian blastocyst and can differentiate spontaneously in-vitro giving rise to pluripotent cells [3]. ESCs can serve as a potential research model for tissue development, cancer formation and metastasis, phenotype commitment, stem cells based therapy, gene therapy strategies and drug design [4]. Despite their clinical potential, the progress of stem cell therapy has been slow, mainly due to issues related to cell quality, phenotype heterogeneity, delivery, integration, proliferation and differentiation inside the host [5].

## Review

### Raman Micro-Spectroscopy (RMS)

During the last century, a plethora of chemical and imaging techniques have been developed to understand the molecular dynamics underpinning the fundamental cellular processes such as proliferation, differentiation or apoptosis [6,7]. Each of these techniques can address certain applications or particular biological problems or processes. Optical microscopy techniques are particularly attractive due to their ability to provide information with high-spatial resolution while using compact and cost-effective instrumentation. Nevertheless, conventional microscopy relies on refractive-index contrast in a sample and does not provide molecular information. Fluorescence microscopy relies on selective imaging of cellular molecules labelled with specific fluorescent dyes [8,9]. Thus, its capabilities are affected by limited stability and photo bleaching of the fluorophores, while the free radical released inside the cells during photo-excitation can induce toxicity. Certain fluorescence labels may influence biochemical processes in cells or cannot enter live cells to access cytoplasmic molecules (often fluorescence imaging requires fixation of the cells).

The Raman effect is based on inelastic scattering of photons when electromagnetic waves interact with molecules. As a consequence of the energy transfer between the vibrating molecules in the sample and the electromagnetic waves, the Raman scattered photons have different frequencies compared to the incident photons, frequencies that are related to the vibrational levels of the molecules. Thus, Raman spectroscopy is a label-free analytical technique and molecular information can be obtained in a non-destructive way with no sample preparation: light is used for excitation of the molecules and the inelastically scattered light is detected for molecular analysis. Raman spectroscopy has been extensively used for molecular analysis of biological tissues and disease diagnostic [10-13].

An important advantage of RMS is that the information regarding molecular vibrations can be obtained using microscopy instrumentation operating in the visible and near-infrared spectral regions. RMS has benefited enormously from the recent advances in high-power laser technology, optical microscopy, fibre optics and detectors with high quantum efficiency in this spectral range. Another important advantage of Raman micro-spectroscopy for live cell imaging is that the background Raman signals from water are significantly weaker compared to signals obtained from cell's molecules. Therefore, RMS can be used to measure repeatedly the molecular properties of live cells maintained in culture media.

### Label-free Raman spectral imaging of cells

The first Raman micro-spectrometer was demonstrated by Dhamelincourt and Delhaye in 1973 [14] but it was not until 1990 when Puppels et al. reported the first Raman spectra of individual cells [15]. Since then, Raman spectroscopy has been extensively used for studies of cells, including detection of protein conformation in living cells [16], chemical differences at different stages of the cell cycle [17,18], differences between cells of different phenotypes [19,20] or apoptotic cells [21]. Raman spectroscopy can probe molecular information from living cells revealing distinct chemical features between nucleus and cytoplasm [15,22-24], lipid structures inside cytoplasm [25], and cell response to diverse stress stimuli [26,27]. High resolution Raman imaging provided new insights into the spatial distribution of chemical species and organelles inside cells [28-34]. RMS was used to follow the cellular uptake of the drugs [35-38] and cellular dynamics [39]. Apoptosis, also known as programmed cell death, is an important biological phenomenon that involves morphological and chemical changes of the cell organelles. Raman spectral mapping of apoptotic cells showed changes in the distribution of the nucleic acids [40,41] and detected the accumulation of lipids when cells were treated with different apoptotic drugs [35,42-44].

Nevertheless, one of the most important features of the Raman spectroscopy is the ability to measure time-course molecular changes in live cells, revealing biochemical processes not attainable with other imaging methods [43-47]. Such experiments are of particular relevance to regenerative medicine and tissue engineering applications as repeated Raman measurements can provide time- and spatially-resolved information regarding the molecular properties of stem cells during differentiation or the growth and development of the engineered grafts. Such information may be used for better understanding the biological processes as well as for improving the bioprocesses.

Raman spectroscopy was also used to study molecular changes at the early stages of differentiation of stem cells [48] and for phenotypic identification of stem cell progeny. The differentiation process describes a series of biochemical changes that are meant to transform a stem cell into a fully functional somatic cell. These changes can be correlated to changes in the Raman spectra of cells measured without using labels or affecting their viability [49]. Recent studies have shown that Raman spectral markers can be used to discriminate fully committed mature cells from undifferentiated cells [23,24,50] and to assess the differentiation status of murine stem cells [51]. In addition, RMS may be used for quality control of cell and tissue grafts prior to clinical use. Recent reports discussed the potential of RMS as a non-invasive method for enrichment of cardiomyocyte populations obtained by differentiation of human stem cells [52]. In this mini-review paper we present three applications of RMS for time- and spatially-resolved molecular characterisation of stem cells during in-vitro differentiation.

### Instrumentation

**Figure 1:**
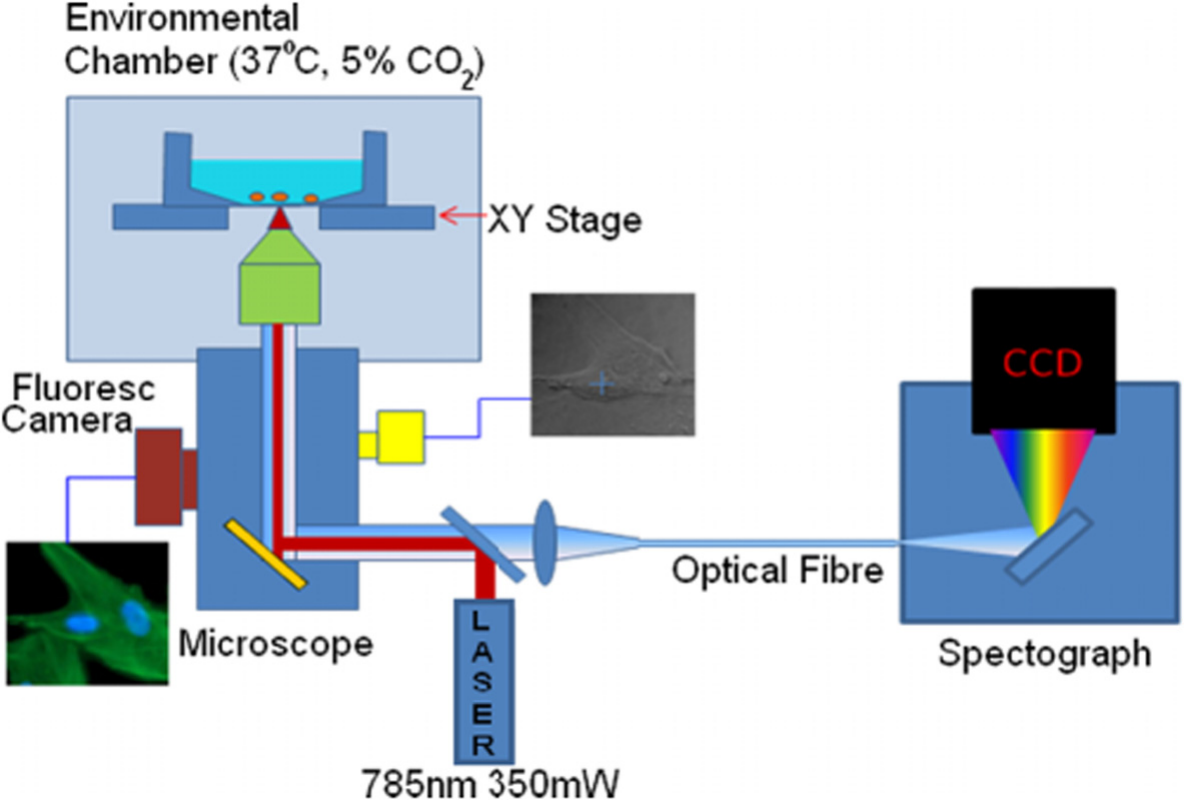
**Schematic description of a Raman micro-spectrometer suitable for monitoring time- and spatially-resolved molecular changes in live stem cells.**

Raman micro-spectroscopy is based on coupling a Raman spectrometer to an optical microscope (Figure 1). Inverted optical microscopes are in particular suitable for time-course cell imaging as the cells can be cultured in dedicated enclosed cell-chambers that allow efficient collection of the Raman scattered light while maintaining the cells in culture media and avoiding bacterial contamination. The instrument used for the applications described in this paper was based on an inverted microscope (IX 71, Olympus, Essex, UK) equipped with an environmental enclosure (Solent, Segensworth, UK) that maintains the live cells at 37°C and 5% CO_2_ atmosphere. A water immersion microscope objective (Olympus) 60×/1.2 N.A. was used to focus the 785 nm laser beam (Toptica Xtra) and collect the Raman scattered light, which was then analysed by an optical spectrometer (830nm diffraction grating, IDus 401 CCD, Andor Technologies, Belfast, UK). The spectrometer was connected to the microscope through a 50 μm diameter optical fibre, providing a spectral resolution of ~4 cm^-1^. A step motorize stage (Prior, Cambridge, UK) was attached to the microscope to raster scan the sample area. The microscope also had an integrated wide-field fluorescence imaging system that allowed imaging of the cells at the end of the Raman spectral measurements, after staining with specific labelles. For typical experiments for cell imaging, the laser power at the sample was in the 100–150 mW range and the exposure time per spot 0.5–2 s (total time required for the aquisiot of a Raman image of a cell ~20–40 minutes at 1 μm step-size).

### Applications

#### Assessment of differentiation status of neuro-progenitor stem cells

Neural stem cells are responsible for the generation of neurons and glial cells and offer great promise to develop treatments for Parkinson’s, Alzheimer’s diseases, chronic inflammatory disorders of the central nervous system, spinal cord injuries or strokes [53]. The capacity of endogenous neural stem cells to proliferate and replace neural cells in-vivo may be affected by chronic inflammatory processes [53]. Hence the repair potential of endogenous stem cells may be limited and any medical procedures designed to mobilize neural stem towards inflammatory sites may be limited. Thus, therapies based on transplantation of neural stem cells from exogenous sources have been developed recently [54].

**Figure 2:**
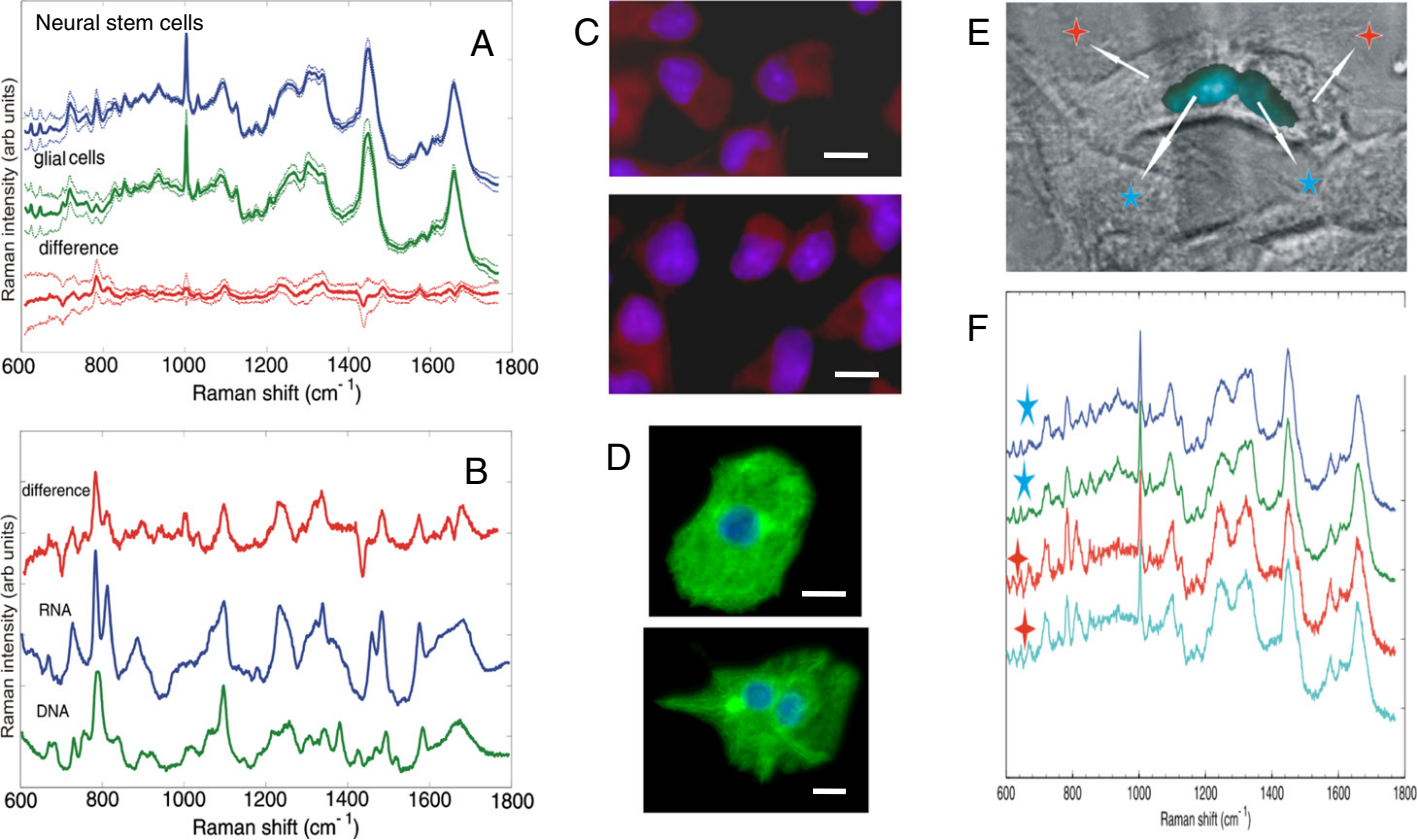
**Analysis of Raman** **spectra of neuroprogenitor and derived-glial cells. (A)** Average Raman spectra of neuro-progenitor stem cells and glial cells, and their computed difference. The side lines represent the standard deviation calculated at each wavenumber. **(B)** Comparison between the computed difference spectrum in **(A)** and the Raman spectra measured from purified RNA and DNA. **(C)** Fluorescence images of undifferentiated neural stem cells (blue=nuclei, red=nestin), **(D)** glial cells (blue=nuclei, green=GFAP) recorded at the end of the Raman measurements. **(E)** Brightfield images of live neuro-progenitor cells indicating the location of the nuclei (fluorescence staining after Raman measurements). **(F)** Raman spectra measured from the live neuro-progenitor cells at locations indicated by the marks. Scale bars: 10 μm.

Raman micro-spectroscopy was proposed as a label-free method for identification of neural stem cells and discrimination of glial cells. Raman spectra of neuro-progenitor cells and glial cells showed significant differences in the fingerprint spectral region (600–1800 cm^-1^) (Figure 2A,B). A multivariate statistical model was developed that allowed discrimination between neural stem cells and glial cells with 89.4% sensitivity and 96.4% specificity [55]. The computed difference between the average Raman spectra of neuro-progenitor and glial cells shows a close similarity with the Raman spectrum of purified RNA (Figure 2B).

Focusing on the 700–830 cm^-1^ region, distinct spectral signatures from nucleic acid base pairs can be observed: adenine (729 cm^-1^), uracil and cytosine (782 cm^-1^ and 785 cm^-1^). Raman bands corresponding to the stretching vibrations of phosphate O-P-O backbone of nucleic acids can also be identified: 788 cm^-1^ for B-DNA, and 788 cm^-1^ and 813 cm^-1^ for RNA [56]. Raman spectra measured at different locations inside live neuro-progenitor stem cells indicate that regions rich in RNA are found mostly in the cytoplasms of these cells (Figure 2E, F).

**Figure 3:**
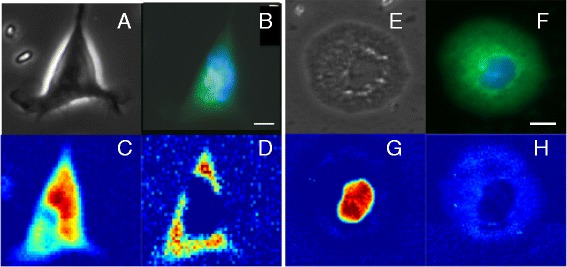
**Raman spectral imaging of neuroprogenitor and derived-glial cells.** Phase contrast images **(A,E)**, DAPI (blue)/FITC (green) fluorescence staining **(B,F)** and Raman spectral images corresponding to the 788cm^-1^ band **(C,G)** and 813 cm^-1^ band **(D,H)** for typical fixed neuroprogenitor and glial cells respectively. (Scale bars: 10 μm).

Raman micro-spectroscopy can measure detailed molecular maps of cells to obtain spatially-resolved chemical information inside the cells with spatial resolution down to the diffraction limit (~750 nm in this case). Figure 3 presents Raman spectral maps along with fluorescence and phase contrast images of neuro-progenitor and glial cells. The Raman maps presented in Figure 3 were constructed by computing the peak area of the 788 cm^-1^ and 813 cm^-1^ bands for each Raman spectrum after subtracting the local baseline. The cells were raster scanned with a step size of 500 nm.

For neuro-progenitor cells (Figure 3), the Raman maps corresponding to the 788 cm^-1^ band is slightly larger than the DAPI staining of the cell nucleus as it contains spectral overlap signature from DNA and RNA. On the other hand, the band at 813 cm^-1^, specifically assigned to RNA, reveals high amount of RNA in the cytoplasm. However, Raman maps recorded for glial cells (Figure 3H) fail to detect bands corresponding to RNA in the cytoplasm. By developing a solution-based calibration model for RNA, the maximum concentration of RNA in the cytoplasm of neuro-progenitor stem cells ranged from 3–5 mg/ml (accuracy in this range is ±0.4 mg/ml) while for glial cells the concentration becomes lower than the detection limit of RNA for our instrument, which is ~1 mg/ml

This finding was somehow surprising considering that the ribosomal RNA represents the dominant type of RNA in cells and most somatic cells have abundant ribosomes. However, the estimates of the cell volumes indicate that the increase in the cytoplasm volume for glial cells by a factor of 4.5 may account for a decrease in the cytoplasm RNA concentration during the differentiation of neuroprogenitor cells to the glial phenotype. The higher intensities of Raman bands corresponding to RNA in neural stem cells may also be related to a higher amount of RNA in the cytoplasm of undifferentiated cells as suggested by earlier histological analysis of embryonic brain explants. These studies showed that neuroepithelial progenitor populations in the ependymal layer have a higher total RNA content than their mature differentiated progeny [57]. Increased concentration of non-translated mRNAs corresponding to the post-transcriptional control of genes has been related to neurogenesis [58], neuronal function [59], as well as stem cell proliferation and embryogenesis [60,61]. For example, high abundance of proteins which repress the translation of mRNAs and maintained the undifferentiated state of neuroprogenitor stem cells have been found in the cytoplasms of these cells [58]. Nevertheless, RMS has the ability to detect and quantify the concentration of RNA in neuroprogenitor cells, with high spatial (less than 1 micron) and temporal resolution (minutes).

#### Time- and spatially-resolved monitoring of mineralisation of bone nodules in-vitro

Bone is one of the largest organs in the human body with numerous mechanical and haematological functions. In addition to age-related diseases, there are congenital bone deformation, bone cancer and bone trauma that may require a bone transplant. Current bone reconstruction and replacement surgical procedures are based on allogeneic tissue grafts. Because of a limited supply of allogeneic bone, tissue engineering and regeneration of the bone based on mesenchymal stem cells has raised a major research interest. The bone grafts obtained in-vitro can be used for clinical applications to restore the functionality of the skeletal system of the patients [1]. The growth of bone in-vitro requires a culture of mesenchymal stem cells (MSCs) in the osteogenic culture medium. Following proliferation and differentiation, MSCs gives rise to the osteoblasts, which are specialized cells responsible for the formation of the bone nodules. Changes during formation and mineralisation of bone nodules can be observed in Raman bands associated to vibrations of hydroxyapatite, mostly in the 950–960 cm^-1^ range [29,30]. RMS was used to record time- and spatially-resolved molecular information during differecntiation of MSCs and formation of bone nodules over 28 days time period.

**Figure 4:**
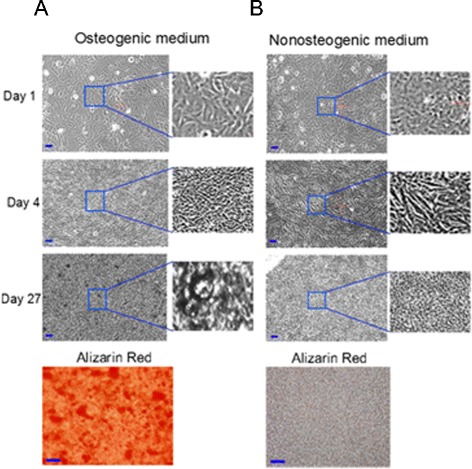
**Time course phase contrast images of mesenchymal stem cells (MSCs). (A)** MSCs cultured in osteogenic media. **(B)** MSCs cultured in control media. Alizarin staining was performed on day 28. Scale bars: 10 μm.

**Figure 5:**
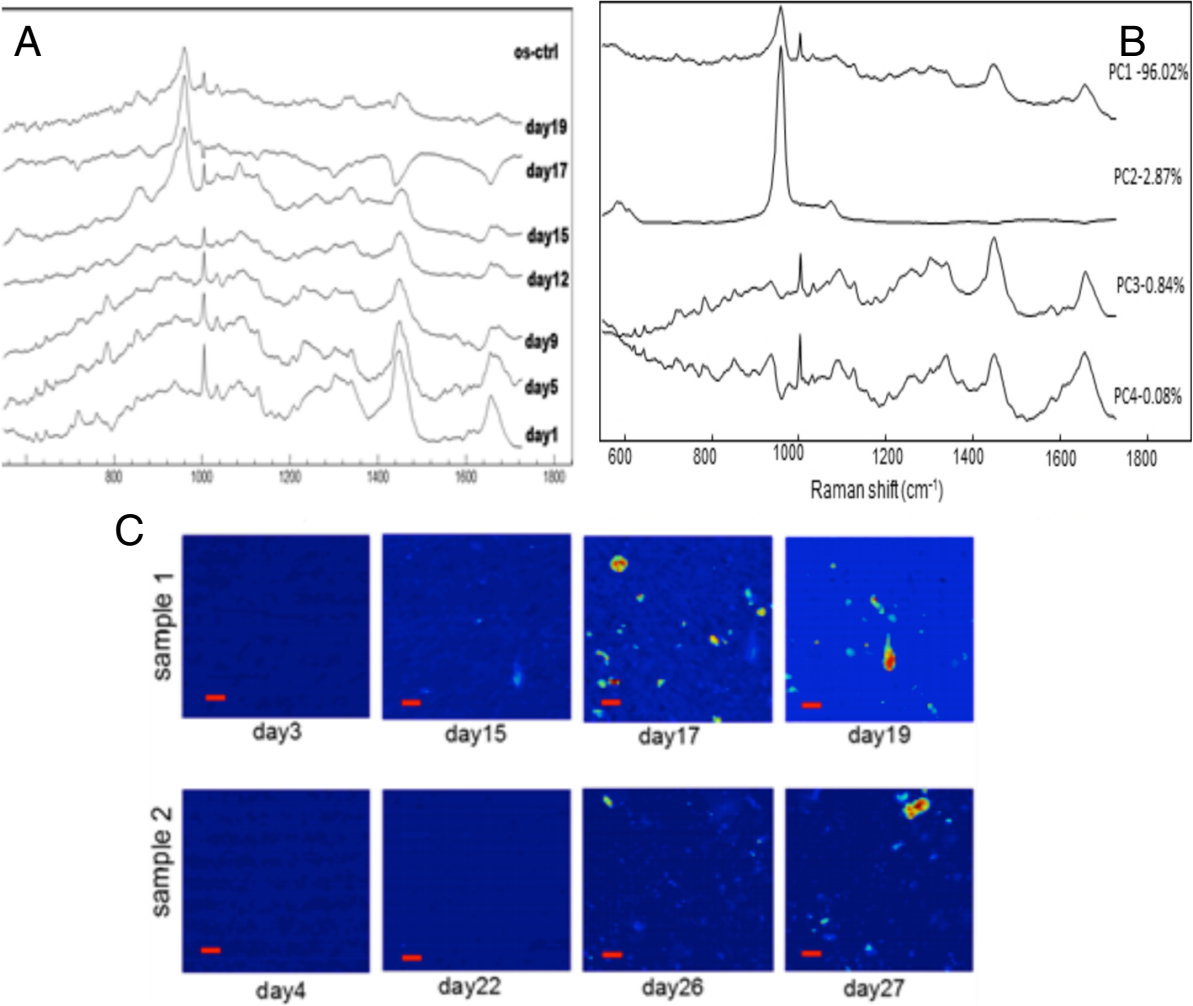
**Time-course Raman spectroscopy of MSC grown in osteogenic medium. (A)** Mean time-course Raman spectra of typical 210 × 210 mm^2^ regions of the cultures shown in C. Raman spectra were acquired at different time points for the same regions of the culture osteoblast in osteogenic medium and non-osteogenic medium. **(B)** Principal components used to extract meaningful chemical information **(C)** Maps corresponding to the PC2 scores recorded in the same culture regions at different measurements days. Scale bar: 10 μm.

Figure 4 presents phase contrast images of MSCs grown in osteogenic and non-osteogenic media at different time-points. Mineralisation of the cultures grown in osteogenic media was confirmed by alizarin red staining performed at the end of the experiments. The difference between Raman spectra of MSCs grown in osteogenic and non-osteogenic media are presented in Figure 5A. Mineralisation of bone nodules is indicated by the strong Raman band at ~959 cm^-1^ corresponding to the PO_3_^−^ symmetric stretching vibrations.

Principal component analysis (PCA) was used to extract temporal and spatial chemical information during differentiation of MSCs. The first four principal components (PCs) containing 99% from the total variance are presented in the Figure 5B. The loading of PC2 resembles the Raman spectrum of hydroxyapatite (HA) [62], which containes a sharp peak at 958 cm^-1^ assigned to PO_3_^−^ symmetric stretching and represents a strong indication of mineralization. Images obtained by plotting the PC2 scores provides information regarding the phase (disordered and crystalline) and spatial distribution of HA. Crystalline HA elicits a sharp Raman band at 958 cm^-1^ while the disordered HA phase is indicated by a broader band in the 940-945 cm^-1^ region. The intensity peak ratio I_958_/I_945_ represents a strong indicator of spatial and temporal evolution of the HA phase (Figure 5C). This ratio was found to increase over time, indicating a higher concentration of disordered phase at the beginning of bone nodule formation leading to a phase transition towards crystalline phase at day 19.

#### Monitoring cardiac differentiation of human embryonic stem cells

The human heart is considered to be a non-regenerative tissue and the permanent loss of cardiomyocytes can lead to cardiac muscle failure. For this reason stem cell therapy is considered a desirable alternative to classical heart transplants for replacing the damaged cardiac tissue. Although these technologies offer great promise for patient with cardiac failure, in-vitro differentiation protocols are not optimised and currently produce cell populations with high phenotypic heterogeneity. Such cell populations require further enrichment and purification prior to clinical use [63].

Pascut *et. al.* showed that RMS can be used for label-free discrimination of individual live cardiomyocytes (CM), derived in-vitro from human embryonic stem cells [64] and investigated the potential for developing Raman-activated cell-sorting of individual cells [52]. A statistical multivariate model using principal component analysis of Raman spectra from stem cell-derived CMs and non-CMs achieved 97% specificity and 96% sensitivity [64]. It was found that the main spectral features that provided the discrimination were related to Raman bands associated to glycogen and proteins [28]. Furthermore, online analysis of the beat frequency of individual cardiomyocytes analysed by RMS showed no significant differences when exposed to the Raman laser, when compared to control cells. In a recent study, RMS was used to monitor the molecular changes in live stem cells during cardiac differentiation, and then to correlate these changes to gold-standard fluorescence staining for the cardiac phenotype [65].

**Figure 6:**
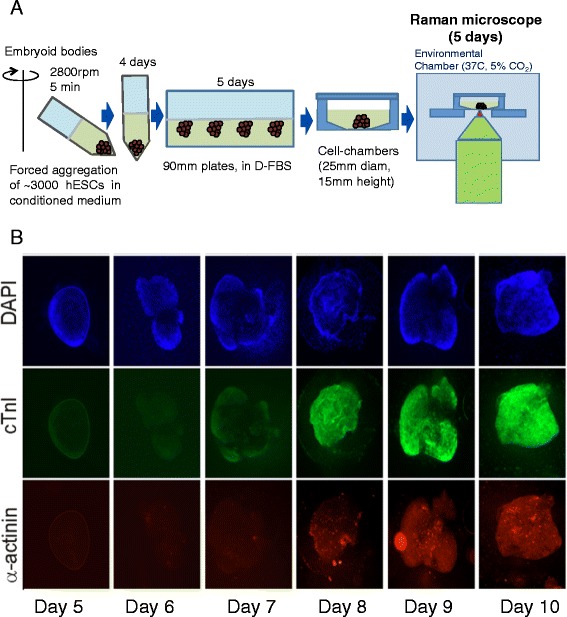
**Time-course Raman spectroscopy measurements on embryoid bodies (EBs) formed by aggregation of human embryonic stem cells. (A)** Schematic description of the time-course measurements. Cells were maintained on the Raman microscope for 5 days, during differentiation days 5–9. **(B)** Immuno-staining of control EBs grown in cardiac medium showing expression of cardiac markers *α* -actinin (red) and cardiac troponin I (cTnI=green) at day 7, corresponding with the onset of spontaneous beating.

Figure 6A presents schematically the design of the experiment in which embryoid bodies (EB) formed by aggregation of human embryonic were grown in conditioned medium on the Raman microscope. The live EBs were grown in conditioned medium to induce differentiation towards cardiac phenotype. Raman spectral maps were acquired by raster-scanning at 24 hour intervals between days 5 and 9 of differentiation, a window wherein cardiac markers were expected to be expressed (immuno-fluorescence on fixed EBs). Flow cytometry and immuno-fluorescence analysis using the CM markers *α*-actinin and/or cardiac troponin I carried out on individual cells dispersed from EBs at day 12 of differentiation indicated that approximately 85% of the cells present in beating EBs were CMs, whereas less than 1% were identified as CM in non-beating EBs.

**Figure 7:**
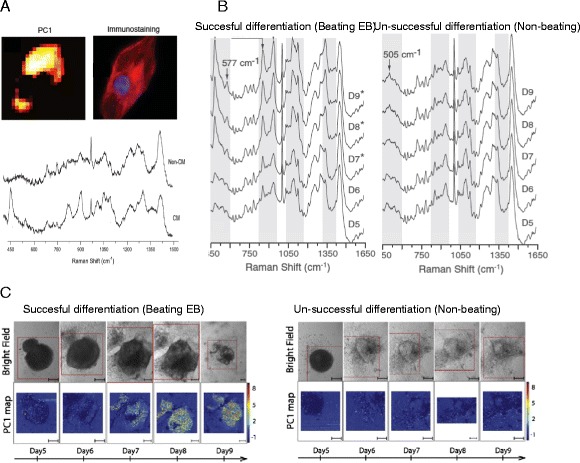
**Raman spectroscopy of cardiomyocytes derived from human embryonic stem cel006Cs. (A)** Raman spectra of beating cardiomyocyte (CM) and non-cardiomyocyte (non-CM) obtained from human embryonic stem cells. PC1 represents the map corresponding to the principal component used for discrimination of CMs and non-CMs in ref [64]. **(B)** Time-course mean Raman spectra of a typical beating and a non-beating EB during days 5–9 (D5–9) of differentiation. *indicates the days at which beating of the EB was observed. **(C)** Raman maps corresponding to PC1 scores for a beating and non-beating EB.

Figure 7 compares the time-course average Raman spectra of typical EBs, beating (successful cardiac differentiation) and non-beating (unsuccessful cardiac differentiation) with the Raman spectra of individual hESC-derived CMs and a non-CM. Significant differences between the Raman spectra of EBs can be observed starting on day 7, when the spectra of the beating EBs showed an increase in the intensity of the bands at 482 cm^-1^, 577 cm^-1^, 858 cm^-1^, 937 cm^-1^, 1083 cm^-1^ and 1340 cm^-1^. The increase in the intensity of these bands was directly correlated with the increase in the number of CMs, and intense bands at the same frequencies can be observed in the Raman spectra of isolated beating CMs derived from hESCs (Figure 7A). These spectral changes were attributed to the formation of myofibrils and accumulation of glycogen in the CMs. These molecular changes are hallmarks for the formation of cardiac tissue and reflect the development of the contractile machinery of the CMs [66,67]. A high accumulation of glycogen in hESC-derived CMs was observed by transmission electron microscopy for CMs derived from several hESCs lines [67] and was related to the increase in fuel demand following the switch from the glycolytic metabolism to oxidative phosphorylaion [68,69].

RMS can also provide time-resolved 2D spectral maps of the EBs and is able to detect the increase in the number of CMs during differentiation. Starting with day 7, the spectral maps show the appearance of CMs in the beating EBs, while no relative increase in the PC1 scores was detected in the non-beating EBs. It was also found that the areas of high PC1 scores also matched the regions of the EBs where the beating was most pronounced, as well as the expression of *α*-actinin obtained by immuno-fluorescence imaging at the end of the time-course experiments [65]. These recent studies show the potential of RMS for non-invasive monitoring of stem cell differentiation, which may enable a more efficient optimization of the relevant bioprocesses.

## Conclusion

This paper reviews recent applications of Raman micro-spectroscopy for time- and spatially-resolved molecular imaging of stem cells during differentiation in-vitro. By integrating the Raman micro-spectrometer with an environmental enclosure, RMS can be used for non-invasive monitoring time-dependent molecular changes in live cells and can provide on-line information regarding the cells and their phenotypic characteristics. RMS may be a useful technique for monitoring bioprocesses and help the refinement and standardisation of differentiation protocols to induce the efficient differentiation of pluripotent stem cells. Such non-invasive techniques are needed to help overcoming the current bottlenecks in the manufacturing and quality assessment of stem cell populations, which are key factors for the future advancement and widespread clinical use of regenerative medicine therapies. In addition, information regarding molecular changes during differentiation can advance the understanding of stem cell differentiation and the development of in vitro models for embryo development. RMS can also provide an invaluable platform for further fundamental studies on stem cells and the effect of various stimuli on their differentiation (eg. mechanical stimulation for osteoblasts), as well as in-vitro testing of new pharmaceuticals on cell models.
